# Protein Acetylation in Archaea, Bacteria, and Eukaryotes

**DOI:** 10.1155/2010/820681

**Published:** 2010-09-16

**Authors:** Jörg Soppa

**Affiliations:** Institute for Molecular Biosciences, Goethe University, Max-von-Laue-StraBe 9, 60438 Frankfurt, Germany

## Abstract

Proteins can be acetylated at the alpha-amino group of the N-terminal amino acid (methionine or the penultimate amino acid after methionine removal) or at the epsilon-amino group of internal lysines. In eukaryotes the majority of proteins are N-terminally acetylated, while this is extremely rare in bacteria. A variety of studies about N-terminal acetylation in archaea have been reported recently, and it was revealed that a considerable fraction of proteins is N-terminally acetylated in haloarchaea and *Sulfolobus*, while this does not seem to apply for methanogenic archaea. Many eukaryotic proteins are modified by differential internal acetylation, which is important for a variety of processes. Until very recently, only two bacterial proteins were known to be acetylation targets, but now 125 acetylation sites are known for *E. coli*. Knowledge about internal acetylation in archaea is extremely limited; only two target proteins are known, only one of which—Alba—was used to study differential acetylation. However, indications accumulate that the degree of internal acetylation of archaeal proteins might be underestimated, and differential acetylation has been shown to be essential for the viability of haloarchaea. Focused proteomic approaches are needed to get an overview of the extent of internal protein acetylation in archaea.

## 1. Introduction

Many different forms of posttranslational modifications of proteins have been characterized. Posttranslational modifications can influence many different features of proteins, for example, their folding, activity, stability, antigenicity, intracellular localization, and interaction with other proteins or with nucleic acids. The fraction of posttranslationally modified proteins and thus the importance of posttranslational modification is generally believed to be very different for eukaryotes—having a high fraction of modified proteins—and prokaryotes, which are thought to harbor only very few modified proteins. 

For eukaryotes it is thought that acetylation is the most common covalent modification out of 200 types that have been reported [1]. It has also been argued that acetylation is a regulatory modification of the same importance as phosphorylation [2]. The arguments were that acetylation, like phosphorylation, affects many different proteins, can have a variety of consequences, and can regulate key cellular processes in response to extracellular signals [2]. Nevertheless, the wealth of experimental data on protein phosphorylation in eukaryotes is much higher than on acetylation, and in addition, it was typically generated earlier. 

In stark contrast to eukaryotic proteins, until very recently only very few bacterial proteins were known to be acetylated. It has long been thought that this would also be true for archaeal proteins, and thus several years ago it was summarized that the available results underscore the conviction that N-terminal acetylation is fundamentally different in eukaryotes compared to archaea and bacteria [3]. The belief that protein acetylation plays an insignificant role in Archaea also held true in the archaeal community for example, in a review about “posttranslation protein modification in Archaea” only about 1% of the text was dedicated to protein acetylation [4]. 

However, results obtained during recent years have revealed that this belief is far from being true and that—in contrast—N-terminal protein acetylation adds to the ever growing number of biological functions that combine eukaryotes and archaea to the exclusion of the bacteria. Earlier recognized examples of similarity between eukaryotic and archaeal systems are the proteins and DNA elements of the transcription initiation machinery [5, 6], the presence of histones [7], the translation initiation factor repertoire [8], and replication proteins [9]. 

However, the information about protein acetylation in archaea is still pretty limited. Therefore, this paper plays a dual role on the one hand it will summarize the results about protein acetylation in archaea, and on the other hand it has the hope to nudge further research in this fascinating and mainly unexplored area. Two different types of protein acetylation are known, which will be discussed sequentially, that is, (1) the acetylation of the alpha-amino group of the N-terminal amino acid of proteins and (2) the acetylation of the epsilon-amino group of internal lysine residues. The former reaction is irreversible, and acetylated proteins stay modified until their degradation, in contrast to the latter, which is reversible and its biological role is thought to be the differential regulation of proteins, for example, the DNA-binding affinity of histones, the most extensively studied example of internally acetylated eukaryotic proteins.

## 2. N-Terminal Protein Acetylation in Eukaryotes and in Bacteria

The occurrence of N-terminal acetylation of proteins in eukaryotes was discovered very early; the first example was described in 1958. In the next decades many examples were found, typically when protein sequencing via the standard method of that time, Edman degradation, was unsuccessful because the N-terminus was “blocked” and alternative methods were developed to unravel the nature of the chemical modification of the alpha amino group. In a review in 1985, already more than 300 examples were listed, which represented more than 100 different proteins, many of which had been analyzed from several species [10]. Recently, techniques have been developed to specifically enrich N-terminal peptides and use mass spectrometric techniques for large-scale analyses of the correct start codon, methionine removal, and covalent modification of the alpha amino group [11, 12]. Examples for their application are the determination of more than 900 N-termini from human HELA cells and of more than 1200 N-termini from *Drosophila melanogaster* proteins [13, 14]. The new large-scale studies underscore that knowledge about N-terminal protein acetylation in eukaryotes reviewed earlier [1, 3] still holds true and give it a higher statistical validation. The identity of the N-terminal amino acid is determined by the activity of the methionine aminopeptidase (MAP). In fact the methionine is removed by MAP from the vast majority of eukaryotic proteins, and thus most proteins start with the second (penultimate) amino acid of the genomically encoded open reading frame. Eukaryotes contain three different N-terminal acetyltransferases (Nat) termed NatA, NatB, and NatC, which have different substrate specificities. They acetylate a high fraction of proteins, for example, around 60% in yeast, 30% in *D. melanogaster,* and more than 80% in humans [14, 15]. NatA acetylates proteins beginning with the N-terminal amino acids Ser, Ala, Gly, or Thr. Therefore NatA depends on the prior action of MAP. As the N-terminal methionine is removed from the majority of proteins, NatA is the major player responsible for N-terminal acetylation. It could be shown that NatA is highly conserved and a yeast NatA mutant could be rescued by human NatA, which acetylated in yeast nearly the same set of proteins as the endogenous NatA, albeit only partially [15]. NatB is specific for proteins starting with methionine and a bulky hydrophobic amino acid in the second (penultimate) position, while NatC is specific for the N-termini Met-Glu and Met-Asp. NatB and NatC are therefore independent of methionine removal by MAP. N-terminal acetylation in eukaryotes occurs cotranslationally, when the amino terminus leaves the ribosome and is complete when the first 40–50 amino acids have been synthesized [3, 16]. In spite of the fact that N-terminal protein acetylation in eukaryotes is known for more than 50 years, the biological function of acetylation of a major fraction of proteins is still not really known. For several proteins, differences between the acetylated and nonacetylated form have been described, including differential activity, biological half life, or thermal stability [3, 17], but a general role of acetylation has not yet been found. An obvious function that has repeatedly been discussed is stabilization of proteins from proteolytic degradation, and the finding that acetylated proteins are strongly overrepresented among the most abundant proteins [18] is in line with this view. However, recently also the opposite has been proposed, namely, that N-terminal acetylation creates specific degradation signals [19]. Irrespective of the clarification of these opposing views, it is clear that N-terminal acetylation is of great significance *in vivo*, because single deletion mutants of all three genes encoding the three Nats in yeast are highly impaired. Characterization of the human homologs has revealed that depletion of hNatB results in a disruption of normal cell cycle progression, while depletion of hNatC induces apoptosis and cell death [20, 21]. 

In stark contrast to the high fraction of N-terminally acetylated proteins in eukaryotes, only very few proteins were found to be N-terminally acetylated in bacteria. Only five *E. coli* proteins are known to be N-terminally acetylated, three of them being the ribosomal proteins S5, S12, and S18. In contrast to eukaryotes, acetylation in *E. coli* occurs posttranslationally. Acetylation of S12 has been shown to stablize the ribosomal stalk complex [22]. Three proteins with Nat activity are known, RimI, RimJ, and RimL. However, at least RimJ has a dual function; a native RimJ as well as an acetylation-deficient variant could suppress the different phenotypes of a ribosomal protein S5 (G28D) mutation, that is, cold-sensitivity, anomalous ribosomal profiles and mRNA misreading. RimJ was found to be associated with the pre-30S subunit, and it was proposed that it is a ribosome assembly factor in addition to being an N-terminal acetyl transferase [23].

## 3. N-Terminal Protein Acetylation in the Archaea

Already 20 years ago it was reported that a few ribosomal proteins of haloarchaea are acetylated at their N-terminus [24–26]. However, this did not increase the interest in protein acetylation in archaea because on the one hand it perfectly fitted to the bacterial paradigm and thus seemed to underscore that protein acetylation is a rare event also in archaea, and on the other hand only extremely few reports about the acetylation of archaeal proteins appeared. The reason why the degree of N-terminal acetylation was overlooked for so long is probably that the acetylation typically is only partial (see below) and that therefore the un-acetylated fraction allowed successful N-terminal sequencing, and therefore ample “proof” was generated that archaeal proteins are blocked in only extreme rare cases. 

One large-scale proteomics study with *Halobacterium salinarum* and *Natronomonas pharaonis *completely changed the picture and currently dominates the view about the degree of N-terminal acetylation in “the archaea.” It should be noted that this is no exception but that the current view about protein acetylation in “the eukaryotes” and “the bacteria” is also based on studies with only one or very few species. The above-mentioned large-scale proteomic study let to the identification of 606 N-terminal peptides from *H. salinarum* and 328 N-terminal peptides from *N. pharaonis*, adding up to a sum of 934 N-termini. On the one hand, the results were used to enhance the reliability of start codon assignments, which is not trivial in high G+C-rich genomes, and to reveal the extent and the rules of N-terminal methionine cleavage in haloarchaea [27]. 

On the other hand, the results were used to specifically address the question of the degree of N-terminal protein acetylation in haloarchaea [28]. It turned out that the N-terminal methionine was cleaved from about two-thirds of all haloarchaeal proteins. Cleavage occurred when the penultimate amino acid was small (glycine, alanine, proline, valine, serine, threonine). The substrate specificity for haloarchaeal methionine aminopeptidases (MAPs) matches the specificity of bacterial and eukaryotic MAPs, and thus the biochemistry of methionine removal appears to be universally conserved. Surprisingly, it was found that N-terminal acetylation is not uncommon in haloarchaea, but that 14% to 19% of the proteins are N-terminally acetylated. 

This is in stark contrast to *E. coli* and reminds more of the situation in eukaryotes; albeit the fraction of acetylated proteins is somewhat smaller. Acetylation occurred nearly exclusively after methionine removal, and only serine and alanine as penultimate amino acids were acetylated [28]. It was found that also the antepenultimate position (position three of the open reading frame and position two in the protein after methionine removal) has a strong influence on protein acetylation. Acetylation is favoured when serine, alanine, and glycine are in the antepenultimate position, while, in contrast, aspartic acid and glutamic acid in this position strongly interfere with acetylation. Therefore, while the degree of acetylation somewhat resembles that of eukaryotic proteins, the substrate specificities of the respective NATs are different as eukaryotic proteins are regularly acetylated when acidic amino acids are in the antepenultimate position [3]. The acetylation pattern is most similar to the substrate specificity of the yeast NatA enzyme, while NatB- and NatC-like activities are missing in haloarchaea. 

Very few exceptions from the “haloarchaeal acetylation rules” were observed, most of which were conserved between both species. Examples are the alpha subunit of the proteasome (see below), the beta subunit of prefoldin and a hypothetical protein. This indicates that in addition to one or more general Nats, also one or very few additional acetyl transferases with a very high substrate specificity exist in haloarchaea. 

It was also shown that the acetylation efficiency for the majority of acetylated proteins is not 100%, but that it differed protein specifically from 13% to 100%. This could be an indication that N-terminal protein acetylation in archaea does not occur cotranslationally, like in eukaryotes, but posttranslatinally. While the genes for putative Nats have been detected in haloarchaeal genomes, as yet no experimental data about the molecular mechanism of N-terminal acetylation are available. 

A few additional proteomic studies enable first estimations about how general these results are true for “the archaea.” A proteomic study with a third haloarchaeon, *Haloferax volcanii*, representing a third genus, mostly underscored the results of the study discussed above [29]. N-terminal peptides of 236 proteins were identified, and the initial methionine was removed in 70% of all cases. 29% of all proteins were N-terminally acetylated, a fraction that is somewhat higher than for the other two haloarchaeal species. One explanation could be that the degree of acetylation is higher in *Haloferax* than in *Halobacterium *and *Nitrosomonas*; an alternative explanation could be that the fraction of identified proteins was lower for *H. volcanii* and that the degree of acetylation is higher for abundant proteins (like in eukaryotes). Nevertheless, the study showed that significant N-terminal acetylation occurs in at least three different haloarchaeal genera and thus can probably be generalized to all haloarchaea.

The situation seems to be different for another group of the Euryarchaeota, the methanogenic archaea. Two proteomic studies are available for *Methanococcus jannaschii*, which identified 72 proteins and 963 proteins, respectively [30, 31]. Only a single acetylation site at an internal lysine was reported [30], while N-terminal acetylation is not mentioned at all. Of course one explanation could be that it was overlooked based on the—at that time—general belief that it does not occur in archaea anyhow. However, I find it more probable to assume that it would have been detected, like in haloarchaea, if it would also occur in methanogenic archaea. Therefore, the two proteomics studies as well as many studies about individual proteins can be taken as an indication that N-terminal acetylation in methanogenic archaea is very rare or does not occur. 

A very limited proteomic survey of N-terminal acetylation was performed with *Sulfolobus solfataricus*, a species belonging to the kingdom of Crenarchaeota [32]. Of the 26 N-terminal peptides identified, 17 were acetylated. Although no “per cent” values can be calculated due to the low absolute numbers of proteins, the fraction of N-terminally acetylated proteins appears to be much higher than for haloarchaea. Clearly a more general study is needed to clarify if these values are of statistical significance. If this would turn out to be true, *Sulfolobales* and maybe Crenarchaeota as a whole would be more alike eukaryotes than all hitherto tested species of Euryarchaeota. For both Cren- and Euryarchaeota it was found that acetylation occurred at penultimate serine and alanine residues, and for both groups partial acetylation was observed. 

The *S. solfataricus* genome was found to contain one gene encoding a putative Nat (sso0209), which was named ssArd1 based on a sequence identity of 37% with the human Ard1, a homologue of yeast NatA. The protein was heterologously produced, and it was confirmed that it can acetylate the N-terminus of Alba, a DNA-binding protein of *Sulfolobus* [32]. A variety of Alba mutants as well as additional *Sulfolobus* proteins were used to characterize the substrate specificity of ssArd1. Surprisingly it was found that in addition to N-terminal Ser and Ala (a NatA-like activity) also the N-termini Met-Glu and Met-Leu were acetylated (NatC- and NatB-like activity). Therefore, it was concluded that the situation in *Sulfolobus* represents an ancestral state with a single Nat, which is not part of a protein complex and which has a broader substrate specificity compared to the eukaryotic Nats. Eukaryotic Nats have later experienced gene duplications and have evolved further into specialized proteins [32]. The fact that some proteins with an N-terminal Ala are not acetylated *in vitro* and *in vivo* was taken as a first indication that N-terminal acetylation in *Sulfolobus* might occur posttranslationally and the degree of folding of the N-terminus determines whether a protein is a substrate for ssArd1.

The exceptional acetylation of the N-terminus Met-Gln was not only found for the alpha subunit of the proteasome in *H. salinarum* and *N. pharaonis* (see above), but also in *Haloferax volcanii* [33]. In the latter species the penultimate, Gln was mutated to several other amino acids, and the consequences for methionine removal and acetylation *in vivo *were characterized [34]. As expected, the introduction of an Ala at the penultimate position resulted in total methionine removal and Ala acetylation, indicating that the protein had been switched from a specific into the default haloarchaeal acetylation pathway. Unexpectedly the N-terminal methionine was not removed in the two mutants Q2S and Q2V, in contrast to the usual substrate specificity of the haloarchaeal MAP. 

Both mutants (Q2S, Q2V) were acetylated at the methionine, indicating that the presumed specific acetylase can tolerate Ser and Val at the penultimate position. Three other mutations (Q2D, Q2P, Q2T) resulted in protein mixtures comprised of methionine cleaved and uncleaved, acetylated and unacetylated forms, indicating that these variants are partial substrates for MAP and specific/default Nat [34]. Clearly, further research is needed to identify the presumed specific Nat and unravel its molecular mechanism. While it does not belong to the topic of this paper, it is interesting to note that the different single amino acid mutants affected the phenotype of the cells, for example, osmotolerance, thermotolerance, or growth rate, underscoring the importance of the proteasome for the physiology of haloarchaea [34]. 

Also the yeast proteasome, which consists of seven different alpha and seven different beta subunits, is target for N-terminal acetylation. In contrast to haloarchaea, no specific Nat is required, but acetylation is performed by the defaults Nats. However, the situation is rather complex: NatA, NatB, and NatC are all required and responsible for the N-terminal acetylation of a specific subset of subunits [35].

## 4. Internal Protein Acetylation in Eukaryotes and Bacteria

In eukaryotes many proteins are differentially acetylated at the epsilon-amino group of internal lysines. Only very little is known about internal protein acetylation in archaea (see below), therefore the overview about internal acetylation in eukaryotes will be kept rather short. By far the most studied eukaryotic target proteins for internal acetylation are the histones, which in addition to acetylation can be posttranslationally modified by phosphorylation, methylation, ubiquitination, and ADP ribosylation [36]. Acetylation shields the charge of the lysine amino group and therefore decreases the binding affinity to DNA; therefore differential acetylation is a means for the regulation of gene expression via differential DNA compaction. Several families of histone acetyl transferases (HATs) and histone deacetylases (HDACs) exist in eukaryotes (e.g., [37, 38]). The names HAT and HDAC are used even when the enzymes have also or even only other targets than histones. Special interest has been given to the Sir2 subfamily of protein deacetylases (also called Sirtuins), which are highly conserved and occur not only in eukaryotes, but also in bacteria and archaea. They are NAD-dependent deacetylases and have been shown to be involved not only in a variety of gene regulatory pathways, but also metabolism, cell motility, multicellular development in social amoeba, longevity in response to caloric restriction, and different kinds of cancers [39–42]. Therefore especially HDACs have been tested as possible targets for anticancer treatments, and HDAC inhibitors have in fact entered Phase I clinical trials [43]. Today many different proteins in addition to histones are known to be regulated by differential acetylation [17, 44] including the cytoskeleton protein tubulin [45, 46]. Acetylation can have an influence on transcriptional regulation, pre-mRNA splicing, protein stability, protein interactions, cell cycle, circadian rhythm, and others [17].

Until very recently it was believed that in bacteria internal acetylation hardly occurs at all. Only two proteins were known to be acetylated at an internal lysine, that is, the chemotaxis protein CheY and the acetyl-CoA synthase. A protein acetyl transferase (PAT) and the deacetylase CobB (belonging to the Sir2 family) have been identified, which regulate the acetylation level of both proteins [47–51]. However, an affinity enrichment of acetylated peptides from *E. coli* led to the identification of 125 acetylation sites in 85 proteins, indicating that internal acetylation is much more common in bacteria than previously thought [52]. The proteins belong to a variety of functional classes, for example, protein synthesis, carbohydrate metabolism, nucleotide metabolism, and TCA cycle. 83 of the 125 acetylation sites were exclusively modified during stationary phase and deacetylated when stationary phase *E. coli* cells were inoculated into fresh medium. Therefore, it has been argued that the main biological function of internal acetylation in *E. coli* might be the downregulation of protein activities during phases of starvation [52].

## 5. Differential Internal Protein Acetylation Is Essential at Least for Haloarchaea

One genetic approach has been performed to elucidate the importance of internal protein acetylation for the haloarchaeal species *H. volcanii* [53]. The genome of *H. volcanii* was found to contain three genes for protein acetylases and two genes for protein deacetylases. All three acetylases belong to the Gcn5 family of acetylases and have been named Pat1, Pat2 (Hvo_1756 and Hvo_1821), and Elp3 (Hvo_2888). One of the deacetylases (Hvo_2194) belongs to the Sir2 subfamily, while the second (Hvo_0522) belongs to the HdaI family. It was attempted to construct single deletion mutants of all five genes. Four of the five mutants could be generated and grew indistinguishably from the wild type. However it turned out to be impossible to generate the *hdaI* deletion mutant, indicating that the deacetylase HdaI is essential for *H. volcanii*. This was experimentally proven by the ability to delete the chromosomal *hdaI* gene in a strain that carried a copy of the gene on a plasmid. As the *hdaI* gene overlaps and is cotranscribed with the gene encoding the histone, it is reasonable to assume that the histone is one substrate for HdaI. It remains to be clarified whether differential histone acetylation is essential for *H. volcanii* or whether the acetylation of other target proteins of HdaI is responsible for the phenotype. While single mutants of all three acetylase genes could be obtained, the *pat2 elp3* double mutant could not be generated. Therefore, the two genes are synthetically lethal indicating that they have overlapping substrate specificities and that the acetylation of at least one protein is essential for *H. volcanii*. The fact that the ability for reversible internal protein acetylation is essential for *H. volcanii* underscores the importance for this posttranslational modification at least for this archaeal species. As genetic techniques for other archaeal species have been developed [54, 55], it will be interesting to clarify whether this is also true for additional species and can be generalized to many or all Archaea.

## 6. Internal Protein Acetylation in the Archaea: ALBA and a Little Bit More

The first archaeal protein with an internally acetylated lysine was reported as early as 1978; it was a 2Fe-2S ferredoxin from *H. salinarum* (at that time named *H. halobium*), which was monoacetylated on lysine 118 near the C-terminus [56]. Shortly thereafter it was reported that the homologous 2Fe-2S ferredoxin from *H. marismortui* (at that time named “*Halobacterium* from the Dead Sea”) was acetylated at the equivalent position [57]. However, these observations did not trigger subsequent interest in differential protein acetylation in archaea and are today known by only few researchers. 

Obvious candidates as targets for internal acetylation are the archaeal histones. It has long been argued that archaeal histones lack the N-terminal domain of eukaryotic histones, which are heavily posttranslationally modified and can therefore not be acetylated. However, meanwhile it has been found that also positions in the conserved histone core domain of eukaryotic histones are acetylated [58]. However, a proteomic approach that specifically addressed the question of histone acetylation in *Methanococcus jannaschii* and *Methanosarcina acetivorans* came to the conclusion that histone acetylation does not occur in either of the two species [59]. Cotranscription of the histone gene with the gene of a deacetylase in *H. volanii* indicates that this might be different in haloarchaea and candidate lysines, which are acetylated in eukaryotes, are conserved in haloarchaea [53], but experimental proof is still missing. 

In the meantime another chromatin protein became the second known target for internal protein acetylation in archaea. It is an *S. solfataricus* protein that had originally been named Sso10b and was renamed “Alba” (acetylation lowers binding affinity) [60]. In this case the observation triggered an intensive characterization of differential acetylation and the responsible enzymes. It was shown that Alba carries two acetyl groups; on the one hand it is N-terminally acetylated, and on the other hand the epsilon-amino group of lysine 16 is acetylated [60]. The *Sulfolobus* member of the Sir2 (Sirtuin) protein family can deacetylate Alba in a NAD-dependent manner. The nonacetylated Alba could repress transcription in an *in vitro* transcription assay, in contrast to the acetylated protein, and the different activities of the two forms were verified after deacetylation of the native protein *in vitro* with Sir2 [60]. The sequence of the *Salmonella* protein acetyl transferase (PAT) was used to identify the *Sulfolobus * homologue, and it was shown that *Sulfolobus* PAT can acetylate Alba *in vitro* with a very high efficiency at the native target amino acid, lysine 16 [61]. The acetylation was shown to reduce the affinity of Alba for double-stranded DNA as well as RNA by a factor of two [61]. The rather moderate influence of the acetylation state of Alba on DNA binding has led to the proposal that it therefore seems probable that the specific acetylation of lysine 16 functions as a modular signal to other proteins rather than as a direct modulation of DNA binding affinity [62]. One interaction partner of Alba is the DNA helicase MCM. It has been shown that Alba strongly inhibits the activity of MCM in an *in vitro *helicase assay. Acetylation of Alba reduced this antagonistic activity of Alba, notably at concentrations at which acetylated Alba bound DNA, excluding an indirect effect [63]. 

While the molecular details of the biological consequences of the different activities of Alba remain to be clarified, it seems to be clear that the acetylation state of Alba influences the degree of “chromatin packaging” in *Sulfolobus*, analogous to the differential acetylation of eukaryotic histones. Structures of Alba from several species are available, and its binding to DNA has been modeled [64–66]. The structure of the Alba acetylase PAT has also been reported [67], and further structures are on their way [68, 69]. In addition, the structure of the deacetylase Sir2 has been determined both in complex with NAD and with an artificial substrate, an acetylated peptide derived from the human protein p53 [70, 71]. Therefore, the archaeal chromatin protein Alba together with its cognate acetylase and deacetylase is by far the best characterized example for the relevance and mechanism of internal acetylation in archaea, and it has the potential to enable the understanding of the molecular mechanism in the near future. 

However, it has been proposed that Alba is not the only acetylated protein in *Sulfolobus* and might not even be the major substrate for PAT and Sir2, because the binding affinity between PAT and Alba is quite low compared to cognate acetylase/protein target pairs in eukaryotes and because PAT is encoded in the genome of species that do not contain Alba [61]. Another argument for the presence of more internally acetylated proteins than currently known is that many archaea encode not only a single but several different protein acetylases and deacetylases. In *H. volcanii,* the protein levels of the protein acetylase Pat1 and the deacetylase Sir2 (both nonessential) have been quantified using specific antisera. It was revealed that Pat1 is constitutively present in the cells while Sir2 is downregulated in stationary phase (Hering and Soppa, unpublished results). This indicates that the acetylation level of proteins increases in stationary phase, reminiscent of the situation that has been described for *E. coli* (see above).

The current situation concerning internal protein acetylation in archaea resembles the situation in bacteria before the first focused large scale study aiming at the identification of acetylated peptides was reported, which increased the number of proven internally acetylated bacterial proteins from two to about ninety. It seems safe to predict that also in archaea many different proteins are differentially acetylated at the epsilon amino groups of internal lysines. Therefore, focused large scale approaches to identify differentially acetylated archaeal proteins are badly needed.

## 7. Novel Approaches to Investigate Protein Acetylation

During recent years several bioinformatic approaches have been developed for a genome-wide prediction of N-terminal acetylation or internal protein acetylation [72–75]. Currently it is unclear whether the rules obtained from eukaryotic proteins can also be used to predict acetylation in archaeal proteomes. For N-terminal acetylation one approach has already been modified using the *H. salinarum* and *N. pharaonis* dataset described above [18]. For internal protein acetylation, benchmarking of bioinformatic programs is another reason why large-scale studies of acetylated lysines are needed. The approach of affinity isolation of peptides with acetylated lysines and their identification by mass spectrometry [52] can and should also be established with a few archaeal species. For the experimental characterization of the influence of acetylation, it would be desirable to be able to compare acetylated and nonacetylated protein variants *in vivo* and *in vitro*. For N-terminal acetylation in *Drosophila,* the (X)PX-rule (proline at first or second position inhibits acetylation) was used to express mutated genes in cells and flies and study the functional relevance of N-terminal acetylation [14]. The authors propose that the (X)PX rule could be applied universally and could be used for equivalent approaches with many other species. For internal acetylation, lysines have often been replaced by other amino acids, but of course in this way not only the acetylation but also the functionality of the lysine is lost. To circumvent this problem, a system has been established for *E. coli* that enables the acetylation of internal lysines in recombinant proteins at any desired position [76].

## 8. Conclusion and Outlook

Knowledge about N-terminal protein acetylation in archaea has increased tremendously in recent years. Several large scale studies are available, and it became obvious that a considerable fraction of proteins is N-terminally acetylated in haloarchaea and in *Sulfolobus*. The situation seems to be different in methanogenic archaea for which N-terminal acetylation has not been mentioned despite the availability of proteomic studies. 

Knowledge about internal acetylation in archaea is still very limited. Only two targets are known, and only one of which has been experimentally characterized (cognate HAT and HDAC, functional consequences). However, there are ample indications that the number of internally acetylated proteins is highly underestimated, including the presence of Pat and Sir2 in species devoid of Alba, the presence of several genes for HATs and HDACs in archaeal genomes, essentiality of HdaI and Pat2/Elp3 in *H. volcanii,* and differential regulation of Sir2 in the same species. The current situation concerning internal acetylation of archaeal proteins resembles the situation concerning *E. coli* proteins before 2008, when the number of known internal acetylation sites was raised from two to 125 due to one proteomic study. Similarly, the role of small noncoding RNAs (sRNAs) for archaeal physiology was unknown a few years ago and today their presence has been proven for any species that was looked at. It can be predicted that differential internal protein acetylation in archaea is another treasure that is waiting to be lifted. It will add an additional layer of complexity to the increasing regulatory network in archaea.
